# A General Mechanism of Green-to-Red Photoconversions of GFP

**DOI:** 10.3389/fmolb.2020.00176

**Published:** 2020-07-29

**Authors:** Dmitry A. Gorbachev, Elizaveta F. Petrusevich, Adil M. Kabylda, Eugene G. Maksimov, Konstantin A. Lukyanov, Alexey M. Bogdanov, Mikhail S. Baranov, Anastasia V. Bochenkova, Alexander S. Mishin

**Affiliations:** ^1^Center of Life Sciences, Skolkovo Institute of Science and Technology, Moscow, Russia; ^2^Department of Chemistry, Lomonosov Moscow State University, Moscow, Russia; ^3^Department of Biophysics, Faculty of Biology, Lomonosov Moscow State University, Moscow, Russia; ^4^Shemyakin-Ovchinnikov Institute of Bioorganic Chemistry, Russian Academy of Sciences, Moscow, Russia; ^5^Institute of Translational Medicine, Pirogov Russian National Research Medical University, Moscow, Russia

**Keywords:** photoconversion, oxidative redding, reductive redding, EGFP, photoinduced electron transfer

## Abstract

Here we dissect the phenomena of oxidative and reductive green-to-red photoconversion of the Green Fluorescent Protein. We characterize distinct orange- and red-emitting forms (λ_abs_/λ_em_ = 490/565 nm; λ_abs_/λ_em_ = 535/600 nm) arising during the Enhanced Green Fluorescent Protein (EGFP) photoconversion under low-oxygen conditions in the presence of reductants. These forms spectroscopically differ from that observed previously in oxidative redding (λ_abs_/λ_em_ = 575/607 nm). We also report on a new green-emitting state (λ_abs_/λ_em_ = 405/525 nm), which is formed upon photoconversion under the low-oxygen conditions. Based on the spectral properties of these forms, their light-independent time evolution, and the high-level computational studies, we provide a structural basis for various photoproducts. Under the low-oxygen conditions, the neutral quinoid-like structure formed via a two-electron oxidation process is found to be a key intermediate and a most likely candidate for the novel green-emitting state of the chromophore. The observed large Stokes shift is traced to the formation of the zwitterionic form of the chromophore in the excited state. Subsequently, this form undergoes two types of cyclization reactions, resulting in the formation of either the orange-emitting state (λ_abs_/λ_em_ = 490/565 nm) or the red-emitting form (λ_abs_/λ_em_ = 535/600 nm). The T65G mutant lacks one of the proposed cyclization pathways and, indeed, the photoconverted T65G EGFP exhibits a single orange-emitting state. In oxidative redding, the red-emitting state resembles the structure of the chromophore from asFP595 (λ_abs_/λ_em_ = 572/595 nm), which is directly formed upon two-electron oxidation and deprotonation bypassing the formation of the quinoid-like structure. Our results disclose a general “oxidative” mechanism of various green-to-red photoconversions of EGFP, providing a link between oxidative redding and the photoconversion under low-oxygen conditions.

## Introduction

The original observations of photoinduced red-shift in the fluorescence emission of the green fluorescent protein in bacteria (Elowitz et al., 1997) under low-oxygen conditions have gained some traction and critique in the literature. This debate was effectively obscured by the discovery of natural red fluorescent proteins (Matz et al., 1999). More than a decade later, a single phenomenon of so-called oxidative redding was characterized in detail for Enhanced Green Fluorescent Protein (EGFP) (Bogdanov et al., 2009b). Contrary to the observations by Elowitz et al. (1997), oxidative redding was described as not sensitive to the presence of oxygen and was attributed to photoinduced electron transfer, facilitated by the presence of oxidants in the solution (Bogdanov et al., 2009b) or even within the immediate amino acid neighborhood of the Green Fluorescent Protein (GFP) chromophore (Bogdanov et al., 2016). The study of oxidative redding has attracted the interest of specialists in quantum chemistry as a model photochemical reaction (Acharya et al., 2017). The interest in the mechanism behind this process was also galvanized by its direct link to the photobleaching of fluorescent proteins (Bogdanov et al., 2009a).

Meanwhile, reports on oxygen-dependent photoconversion of green fluorescent proteins in living systems, such as yeast (Jakobs et al., 2003) have continued to appear in the literature, crowned by the demonstration of photoconversion of EGFP from green to red form facilitated by reduced flavin (Matsuda et al., 2010). Later reports (Ai et al., 2015) indicate green-to-red photoconversion (enhanced in a low oxygen environment) of GFP-based genetically encoded sensor GCaMP in insect and mammalian cells. The utility of green-to-red GFP photoconversion was shown for transgenic plants, insect and mammalian cells *in vivo* (Sattarzadeh et al., 2015). In this work, we sought to dissect two potentially intertwined and possibly mixed phenomena behind conflicting reports of oxygen-dependent and oxygen-independent green-to-red photoconversions of the green fluorescent protein.

## Materials and Methods

### Protein Expression and Purification

For protein expression, the coding sequences of fluorescent proteins were cloned in the plasmid pQE-30 (Invitrogen, United States).

The fluorescent protein denoted as EGFP in this work has the following amino acid sequence:

>EGFP

MVSKGEELFTGVVPILVELDGDVNGHKFSVSGEGEGDAT YGKLTLKFICTTGKLPVPWPTLVTTLTYGVQCFSRYPDHMK QHDFFKSAMPEGYVQERTIFFKDDGNYKTRAEVKFEGDTL VNRIELKGIDFKEDGNILGHKLEYNYNSHNVYIMADKQKN GIKVNFKIRHNIEDGSVQLADHYQQNTPIGDGPVLLPDNH YLSTQSALSKDPNEKRDHMVLLEFVTAAGITLGMDELYK

The protein denoted as EGFP-T65G carries X = Gly instead of X = Thr in the chromophore-forming tripeptide (-X-Y-G-).

Fluorescent proteins were expressed in XL1 Blue *Escherichia coli* strain (Invitrogen, United States). The single bacterial colony was inoculated into 200 ml of LB medium with IPTG and ampicillin (1 mM and 200 mg/ml, respectively). The flasks were then incubated at 37°C with vigorous shaking (250 rpm). After 12 h 100 ml LB media with 10 mM IPTG and 200 mg/ml ampicillin was added in the culture and grown for 6–8 h.

The culture was centrifuged, the supernatant was discarded, the pellet was resuspended in 5 ml of phosphate buffer (pH 7.4), the suspension was lysed in Sonics Vibra Cell sonicator (Sonics & Materials, Inc., United States). The cell lysates were cleared by centrifugation (16000 *g*, 10 min, 4°C) and incubated with TALON Metal Affinity Resin (Takara Bio Europe S.A.S.) for 15 min. The resin was washed with PBS with 10 volumes of the column. The protein was eluted with PBS containing 100 mM EDTA (pH 7.4). Finally, all purified samples were concentrated and equilibrated in PBS (pH 7.4). Sodium dodecyl sulfate-polyacrylamide gel electrophoresis(SDS-PAGE) analysis was performed in denaturing conditions according to standard protocols in 12.5% polyacrylamide gel.

### Photoconversion

The cuvette with a 2 μM of fluorescent protein in 1 ml PBS solution was illuminated for 1 (for oxidative redding) or 5 (for reductive redding) minutes with focused blue light from seven ultra-bright light emitting diodes (LXML-PB02 LUXEON Rebel high power LED, 470 nm, Luxeon Star LEDs Quadica Developments, Inc., Canada). The light was focused (600 mW/cm^2^) on the cuvette, and the entire apparatus was covered in light-reflective aluminum foil. We assessed photoconversion of fluorescent proteins in two major experimental conditions – those in favor of oxidative redding, containing strong oxidants described in the original paper (Bogdanov et al., 2009b), or in favor of reductive photoconversion.

For reductive photoconversion, we selected sodium dithionite (Na_2_S_2_O_4_ was added to phosphate buffer saline, pH 7.4, to the final concentration of 10 mM). Sodium dithionite reacts with the dissolved oxygen, therefore serving as both reductant and an oxygen scavenger. Alternatively, we used slow bubbling (for ∼15 min) of oxygen-free argon through the buffer to remove dissolved oxygen. In this case, active reductant was produced in the reaction of the photoreduction of FAD in the presence of EDTA (Penzer and Radda, 1968). A similar photoreduction of flavins was used in previous reports of green-to-red photoconversion of GFP. We used an excess of 10 mM EDTA and the FAD concentration on the same scale (5 μM) as the concentration of the fluorescent protein (2 μM). All results in this study were reproduced in both conditions, except for the fluorescence lifetime estimation, which was performed with sodium dithionite only.

For oxidative photoconversion, we followed the protocol described previously (Bogdanov et al., 2009b). In the current study, the protein sample was dissolved in the 50 mM phosphate buffer saline, pH 7.4, and irradiated by 470 nm light in the presence of 10 mM K_3_[Fe(CN)_6_].

### pH Titration

For the measurement of pH dependence of the fluorescence spectra, we used 50 mM buffers in the pH range from 3.5 to 11 (citrate buffers in the pH range 3.5–4.5, phosphate buffers in the pH range 5.0–7.5, and borate buffers in the pH range 8.0–11.0). An aliquot of protein sample after photoconversion was diluted in the corresponding buffer solution and immediately placed in the spectrophotometer for measurements.

### SDS-PAGE

SDS-PAGE analysis was performed using a 12% polyacrylamide gel. A 25 μg aliquot of protein was added to the loading buffer mix and adjusted to a total of 50 μl. The solutions were mixed well and heated to 100°C for 5 min in a PCR machine. 5 μg of protein (10 μl) was loaded to each lane of the gel. Electrophoresis was performed in the SDS-Tris-glycine buffer. The voltage for the concentrating gel was set to 100 V, and to 200 V for the separating gel. The gel was stained with Coomassie dye blue diamond G-250.

### Spectroscopy

Varian Cary Eclipse Fluorescence spectrophotometer was used to measure excitation and emission spectra. The absorbance spectra were acquired with Cary 100 UV/VIS spectrophotometer.

Fluorescence-decay kinetics was collected by a time- and wavelength-correlated single-photon counting setup (Becker & Hickl GmbH, Berlin, Germany). The temperature of the sample was stabilized at 21°C by a Peltier-controlled cuvette holder Qpod 2e (Quantum Northwest, Inc., Liberty Lake, WA, United States) with a magnetic stirrer. 2 min irradiation of the sample with focused light from 200 mW royal blue (central wavelength 455 nm) LED (Thorlabs) was used for photoconversion. Excitation of EGFP was performed with ps-pulsed lasers at either 405 or 510 nm (InTop, Russia), driven at a repetition rate of up to 50 MHz. Longpass filter 510LP (Thorlabs) was used to filter out excitation light. A monochromator additionally filtered the emission (ML-44, Solar, Belarus) set to either 610 or 525 nm (21 nm FWHM). Finally, the emission was collected with a photon-counting detector (HPM-100-07C, Becker & Hickl GmbH, Germany) with a low dark count rate (∼10 counts per second). SPCImage (Becker & Hickl, Germany) software package was used for approximation of fluorescence decay curves by a sum of exponential decay functions.

### Computational Details

Atomistic models of various forms of the S65T-GFP protein were constructed based on the X-ray data [PDB ID: 1EMA (Ormö et al., 1996)]. The E222 residue was kept protonated, while the initial green GFP chromophore was treated as the anion. All histidine residues were taken uncharged and δ-protonated. The overall net charge of the protein was set to zero by performing point modifications of charged amino acid residues exposed to solvent to their neutral analogs (D19N, E32Q, E34Q, D117N, and D173N) along with the C-terminal amino acid residue that was kept neutral. The protein model was then placed in an aqueous cell containing approximately 6900 water molecules. The size of the resulting system was 54 Å 57 Å 70 Å, which contained approximately 23000 atoms. Constant temperature NVT (the canonical statistical ensemble with three fixed parameters, the number of particles in the system N, the system’s volume V, and the system’s absolute temperature T) molecular dynamics simulations with the CHARMM force field parameters (MacKerell et al., 1998) were performed using the Langevin thermostat at 300 K for 2 ns with an integration step of 1 femtoseconds (fs). Periodic boundary conditions were applied. Long-range electrostatic interactions were treated with the particle mesh Ewald method. After equilibration at room temperature, the system was gradually cooled down to 50 K in steps of 50 K during 1.2 ns. The protein backbone and the chromophore were held fixed during the simulation. The full atomistic model of the S65T GFP protein was constructed using Visual Molecular Dynamics (VMD) (Humphrey et al., 1996) and NAMD (Phillips et al., 2005).

The final molecular dynamics (MD) structure was reduced for the subsequent quantum mechanical/molecular mechanical (method) (QM/MM) calculations by removing outer water molecules located farther than 2.9 Å from the protein. The total number of atoms in the initial green form of the S65T GFP protein was equal to 5410. The quantum mechanical (QM) part (154 atoms) included the chromophore and all nearby amino acid residues (Glu222, His148, Ser205, Arg96, His148, Ser205). Ground-state equilibrium geometry parameters were obtained at the PBE0/(aug)-cc-pVDZ//CHARMM level of theory using a mechanical embedding QM/MM scheme. All residues within 7 Å from the QM region were optimized, whereas the remaining MM residues were held fixed during the geometry optimization. QM/MM partial Hessian evaluation and vibrational analysis were performed to ensure that located stationary points were true minima. The details of the computational approach can be found elsewhere (Bochenkova and Andersen, 2013).

Vertical excitation energies (VEE) were calculated using extended multiconfiguration quasi-degenerate perturbation theory XMCQDPT2 (Granovsky, 2011) with model spaces spanned by two or more state-averaged CASSCF zero-order wave functions and active spaces including π orbitals. The (aug)-cc-pVDZ basis set was used. The reference states of the anionic green and red singlet forms were obtained using SA(2)-CASSCF(16,14) and SA(2)-CASSCF(16,15) wave functions, respectively. The neutral singlet (quinoid-like green and orange structures), open-shell neutral radical and anionic triplet forms were treated using larger dimensions of reference spaces spanned by all relevant zeroth-order SA(7)-CASSCF(14,14), SA(7)-CASSCF(15,14), and SA(9)-CASSCF(16,14) functions, respectively. Larger reference spaces ensured a correct ordering of states after diagonalization of effective Hamiltonians calculated perturbatively and resulted in stable solutions for target electronic states saturated within the chosen active spaces. The protein environment was represented by a set of MM point charges and included in the electronic Hamiltonian using the effective fragment potential (EFP) method (Day et al., 1996), allowing the QM electron densities of the ground and excited states to be polarized by the protein field. For all electronic structure calculations, the Firefly computational package, version 8.2 (Granovsky, 2016), partially based on the GAMESS (United States) source code (Schmidt et al., 1993), and its modified QM/MM versions were used.

## Results

### Establishing Conditions for Robust Green-to-Red Photoconversion in the Absence of Oxygen

**FIGURE 1:**
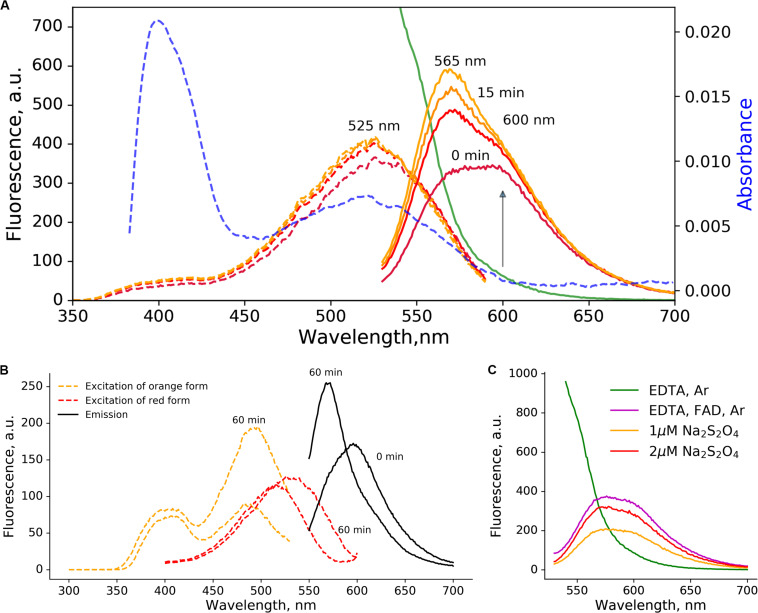
Orange and red-emitting photoproducts of low-oxygen photoconversion of EGFP. The 2 μM solution of EGFP in the presence of sodium dithionite (10 mM) was illuminated for 5 min with blue light (470 nm, 600 mW/cm^2^). **(A)** Fluorescence emission spectra (excitation at 520 nm, solid lines): green – before the photoconversion, from purple to yellow – 0, 5, 10, and 15 min after the photoconversion. The blue dashed line – absorbance after the photoconversion. The purple-to-orange dashed lines – excitation spectra (emission at 620 nm), color-coded similarly to the emission spectra. **(B)** changes in excitation (dashed lines, orange – for emission at 540 nm, red – for emission at 610 nm) and emission (solid black line, excitation at 540 nm) spectra with time; **(C)** emission spectra (excitation at 520 nm) of irradiated EGFP samples (5 min, 470 nm, 600 mW/cm^2^). In the absence of reductants (no FAD, green line) no red-emitting forms are visible even in low-oxygen conditions induced by Ar bubbling. Addition of FAD (magenta line, 10 mM EDTA, 5 μM FAD) or sodium dithionite (orange and red lines) without Ar treatment results in similar spectra.

First, we established conditions for *in vitro* green-to-red photoconversion of EGFP with lowered oxygen. We followed experimental design similar to one in the report by Matsuda et al. (2010), which was based on the photoreduction of flavins by amino acids (Penzer and Radda, 1968) under the same blue light illumination that drives the photoconversion of EGFP. We replaced riboflavin with FAD due to direct indications of the involvement of riboflavin in oxidative redding (Bogdanov et al., 2012). To avoid the use of the FAD-containing enzymatic oxygen scavenging system (glucose oxidase), we removed the oxygen by slow bubbling of argon immediately before the experiment. The spectroscopic cuvette was then sealed tight and illuminated with blue light (∼470 nm). We also lowered the concentration of flavins 10-fold in comparison with the conditions used by Matsuda et al. (2010) to reduce further the risks of competing oxidative redding process, which typically occurs in the presence of tens of micromoles of oxidants (Bogdanov et al., 2009b). Finally, we achieved efficient photoconversion of EGFP (2 μM) in the presence of EDTA (10 mM), and FAD (5 μM). No photoconversion was observed in the presence of oxygen or in the absence of either EDTA or FAD. We achieved similar efficiency of the photoconversion by dissolving sodium dithionite (final concentration of 10 mM Na_2_S_2_O_4_) in the sample, without additional steps for oxygen removal due to intrinsic oxygen-scavenging activity of sodium dithionite (Figure 1C). The cuvettes were filled to the brim and sealed tight immediately after addition of sodium dithionite in order to prevent contact with the atmospheric oxygen.

### Spectral Properties of Orange- and Red-Emitting States Arising in the Course of the Photoconversion Under Low-Oxygen Conditions

The photoconversion of EGFP in low-oxygen conditions resulted in two distinct red-emitting forms with emission maxima at 600 and 565 nm. Immediately after the photoconversion, the 600 nm form was prevalent, while another form with emission maxima 565 nm emerged in a light-independent manner within minutes (Figure 1A).

The single broad absorbance peak with the maximum at 525 nm was observed, which engulfs peak in the excitation spectra of both red-emitting forms. We followed the light-independent changes in the excitation and emission spectra (Figure 1B), and observed a rise in ∼490 nm peak, which coincides with the increase in orange (565 nm) emission. Therefore, we attributed 490 nm to the excitation maximum of orange-emitting form (565 nm), and the 535 nm to the excitation maximum of red-emitting form (600 nm).

### Green-Emitting Form With a Large Stokes Shift Is Formed in the Course of “Reductive” Photoconversion

**FIGURE 2:**
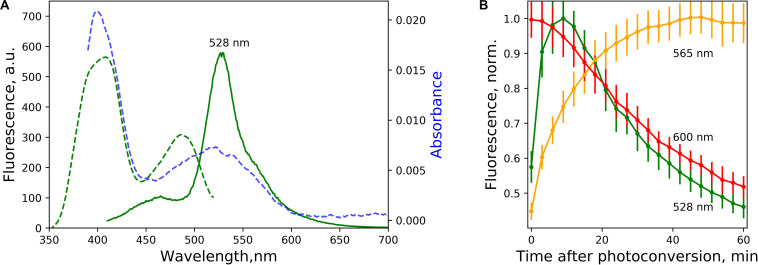
Green-emitting forms and the time course of light-independent interconversion of spectral species. **(A)** Excitation (green dashed line, emission at 530 nm) and emission (green solid line, excitation at 400 nm) spectra of green-emitting form 5 min after photoconversion. Blue dashed line – absorbance after the photoconversion. **(B)** Time course of light-independent fluorescence change of the forms with emission at 525 nm (excited at 400 nm), 565 nm, and 600 nm (excited at 525 nm) after the photoconversion, pH = 7.4. The lines represent the mean value, error bars – standard deviation (*n* = 3 independent photoconversions).

We noted a ∼400 nm peak in the absorbance spectra after photoconversion. Surprisingly, upon excitation of the EGFP solution at 400 nm after the photoconversion in low-oxygen conditions (either with EDTA+FAD or sodium dithionite), we observed a distinct peak with green emission peaked at 525 nm, red-shifted in comparison with the emission spectra of EGFP (Figure 2A). Apparently, the existence of this spectral form was overlooked in previous works due to the overlap with EGFP spectra and incomplete photoconversion. Indeed, we observed a prevalence of 400 and 525 nm absorbance over 488 nm centered absorbance peak of EGFP after 5 min of photoconversion (Figure 2A, blue dashed line), while the low yield of photoconversion precluded such measurements in the original report (Elowitz et al., 1997).

### Time Course of Light-Independent Change in Fluorescence Spectra in the Course of “Reductive” Photoconversion

We followed the evolution of the emission of all three distinct spectral forms associated with the photoconversion of EGFP in low-oxygen conditions (Figure 2B). The fluorescence of red form with emission peaked at 565 nm increased steadily for tens of minutes after the photoconversion, while both 600 nm peaked and 525 nm peaked forms eventually decayed.

**FIGURE 3:**
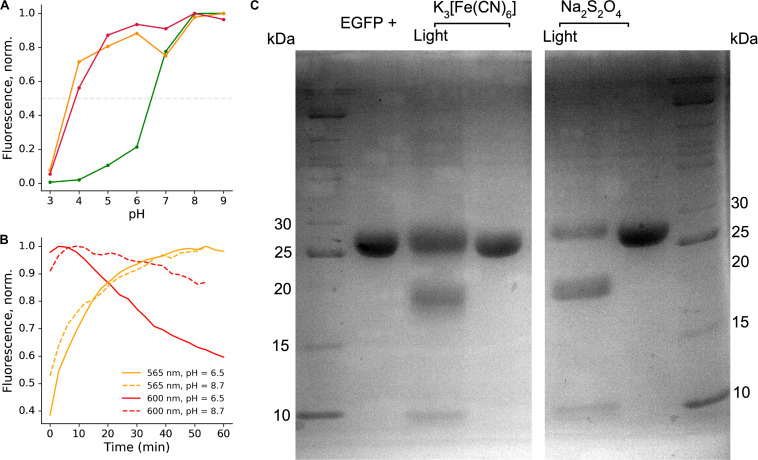
Stability of the EGFP photoproducts. **(A)** Representative pH titration curve of the photoproducts in the presence of sodium dithionite; green line – emission at 525 nm, orange line – emission at 565 nm, red line – emission at 600 nm. **(B)** Representative time course of the light-independent stage of the photoconversion at different pH, showing better stability of the red-emitting form at high pH. **(C)** SDS-PAGE analysis of the light-irradiated and untreated EGFP samples in the presence of potassium ferricyanide or sodium dithionite.

We estimated the apparent pKa of green-emitting form as ∼6.5 (Figure 3A). We measured apparent pKa for 565 nm and 600-nm emitting forms, with indistinguishable value for both forms of ∼3.5 (Figure 3A). Notably, the red-emitting (600 nm) form appeared to be more stable with the increase in the pH value (Figure 3B).

Backbone fragmentation is observed in SDS-PAGE of protein samples after photoconversion.

Fluorescent proteins harboring labile bonds within the protein backbone (such as acylimine) are typically unstable in conditions of SDS-PAGE, as was observed for DsRed-like chromophores (Gross et al., 2000). Therefore, we performed SDS-PAGE analysis of EGFP before and after photoconversion with either potassium ferricyanide or sodium dithionite. The protein fragments indicating cleavage near the chromophore appeared in both types of photoconversion (Figure 3C). Apparent masses were in agreement with calculated masses of the fragments (8 + 19.7 kDa, taking into account 6xHis tag).

### Time-Resolved Spectroscopy of Photoproducts of EGFP Photoconversions

**FIGURE 4:**
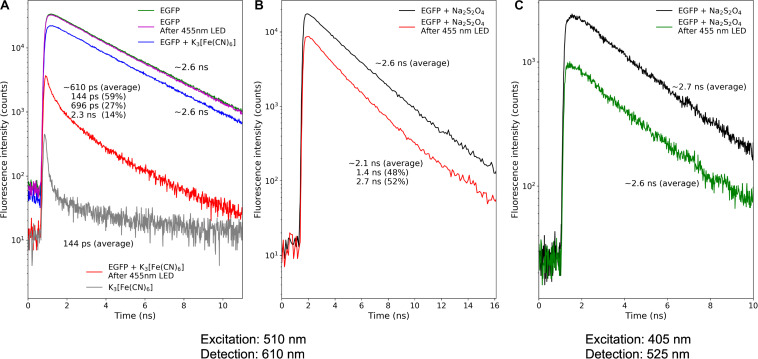
Fluorescence decay kinetics of the EGFP photoproducts. **(A)** Photoconversion in the presence of potassium ferricyanide; **(B,C)** photoconversion in the presence of sodium dithionite. Repetition rate of pulsed light sources: 50 MHz **(A)** and 10 MHz **(B,C)**.

Due to the lack of fluorescence lifetime data on EGFP photoconversion *in vitro* in the literature, we performed time-resolved measurements. Two excitation/detection settings were used: 405/535 and 510/610 nm (Figure 4).

For the oxidative redding, we measured the fluorescence lifetime of the EGFP solution immediately after photoconversion with a 455 nm LED light source in the presence of potassium ferricyanide (10 mM). We estimated the lifetime of red-emitting form arising in the course of oxidative redding of EGFP as ∼0.6 ns.

Similarly, we measured the fluorescence lifetime of the photoproducts of EGFP photoconversion in the presence of sodium dithionite. The mean lifetime of the red emission upon 510 nm pulsed laser excitation was ∼2.1 ns. We exploited the light-independent increase of the fraction of the 565 nm-emitting form to unmix the observed lifetime to two distinct components: ∼1.4 and ∼2.7 ns, the amplitude of the component with the lifetime of 1.4 ns was rising in agreement with the increase of the 565 nm emission in the steady-state fluorescence emission spectra. Therefore we attributed the 1.4 ns component to the fluorescence lifetime of 565-nm emitting form and the 2.7 ns component to the form with an emission maximum at 600 nm. We further estimated the mean fluorescence lifetime of green-emitting form as ∼2.6 ns.

### Photoconversion of Mutant Protein Harboring T65G Mutation Results in Orange-Emitting Form

**FIGURE 5:**
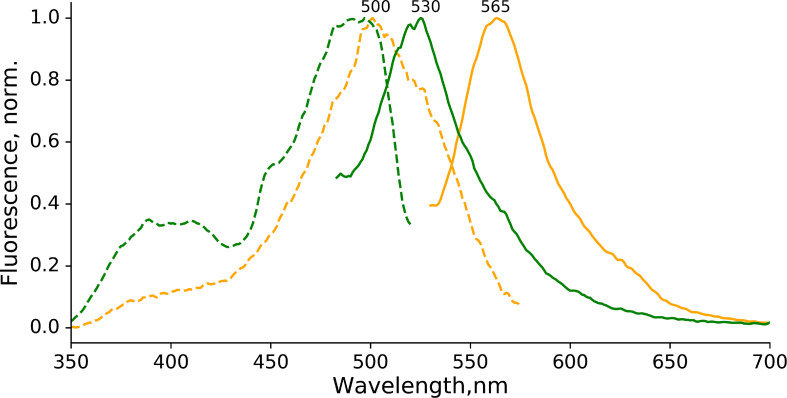
Photoconversion of EGFP-T65G in the presence of sodium dithionite. Orange form: excitation (dashed line, emission at 600 nm) and emission (solid line, excitation at 500 nm) spectra immediately after photoconversion). Green form: excitation (dashed line, emission at 600 nm) and emission (solid line, excitation at 400 nm) spectra immediately after photoconversion). The conditions for photoconversion are similar to the ones used for EGFP.

The first position in the conservative chromophore-forming triade (-X-Y-G-) is typically restricted to a limited set of amino acids in orange-emitting fluorescent proteins, namely Thr, Lys, Ser, or Cys (Pletnev et al., 2014). We therefore assessed the photoconversion of EGFP-T65G in the presence of sodium dithionite. Surprisingly, we detected the orange-emitting form (Figure 5) almost indistinguishable from the one observed for EGFP (excitation maximum 500 nm, emission maximum 565 nm). Notably, the red-emitting form (with expected excitation ∼535 nm and emission ∼600 nm) was not detected in the photoproducts of the photoconversion of EGFP-T65G.

### Calculated Ground-State Structures of Various Spectral Forms and Their Vertical Excitation Energies

High-level *ab initio* calculations have been used to characterize various spectral forms of the chromophore inside the S65T GFP protein and to aid the interpretation of the experimental results. The green form has been obtained previously (Bochenkova and Andersen, 2013). The calculated VEE (493 nm) is well consistent with the experimental absorption maximum at 489 nm (Ormö et al., 1996), thus validating the computational methodology used in the present study.

Originally, the structure of the DsRed-like chromophore, which can be formed as a result of a two-electron oxidation process, has been proposed as a red-emitting form in oxidative redding (Bogdanov et al., 2009b). The DsRed-like chromophore and its close analog with the cleaved peptide bond, the asFP595-like chromophore, have been thus considered as most likely candidates for the red-emitting form observed upon oxidative photoconversion.

**FIGURE 6:**
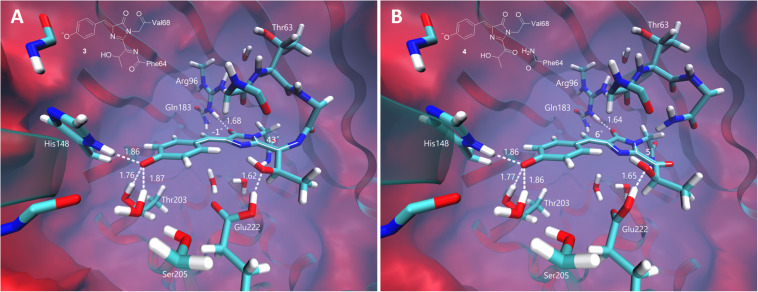
Ground-state equilibrium structures of the red-emitting forms. **(A)** The non-hydrolyzed DsRed-like chromophore. **(B)** The hydrolyzed asFP595-like chromophore. Only the QM parts are shown for clarity. All distances are shown in Å.

The equilibrium structures of the red-emitting forms similar to those from the DsRed and asFP595 proteins are shown in Figure 6. The DsRed-like chromophore exhibits a non-planar structure inside the protein. The imidazolinone and phenol rings are nearly coplanar; however, the newly formed CN double bond deviates from the plane of the conjugated system by 43°. The structure of the hydrolyzed form, similar to that of the asFP595 chromophore, adopts an almost planar structure due to the cleavage of the peptide bond. According to this, the calculated VEE of the asFP595-like chromophore is red-shifted (580 nm) compared to that of the DsRed-like chromophore (565 nm). Both transitions are bright, with an oscillator strength of ∼0.9. The experimental absorption maxima of the DsRed and asFP595 proteins are 558 (Matz et al., 1999) and 572 nm (Lukyanov et al., 2000), respectively. These values are consistent with our calculations, suggesting that the red-emitting form observed in oxidative green-to-red photoconversion (absorption 575 nm, emission 607 nm) can be attributed to one of these structures, and, in particular, to the most red-shifted planar structure of the asFP595 chromophore.

**FIGURE 7:**
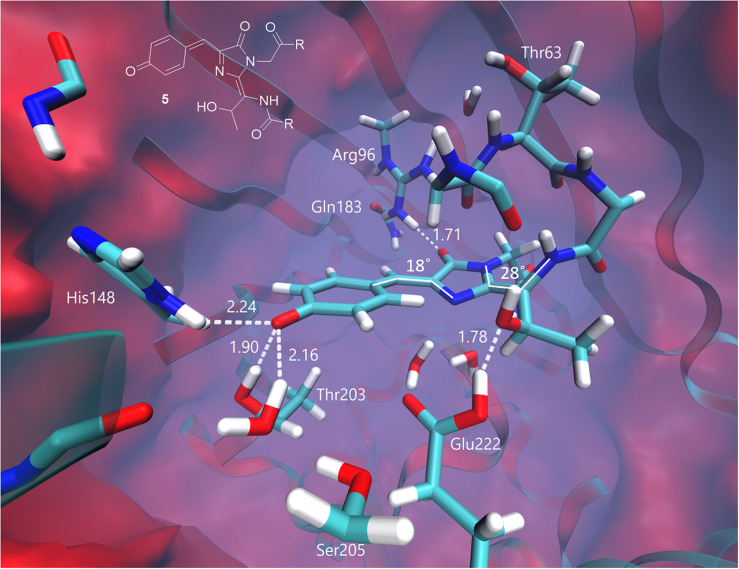
Ground-state equilibrium structure of the quinoid-like form. Only the QM part is shown for clarity. All distances are shown in Å.

At the same time, the green form of the chromophore transforms into the neutral quinoid-like structure with an extended π-conjugated system upon two-electron oxidation and single deprotonation. The equilibrium structure of this form is shown in Figure 7. The planarity between the imidazolinone and phenol rings is disturbed in the chromophore, partially disrupting the conjugation in the system. The angle between the CN bond that undergoes changes during photooxidation and the plane of the imidazolinone ring is 28°. Distortion of the planarity in combination with the neutrality of the chromophore leads to a hypsochromic shift of the vertical transition energy relative to those of other structures. The calculated VEE is 406 nm, with an oscillator strength of 0.52. This value is consistent with the absorption at around 400 nm, corresponding to the green-emitting form observed in the photoconversion under the low-oxygen conditions.

The experimental emission maximum of this form is 525 nm. According to our calculations, the ground-state electronic structure of this form is best described as the neutral quinoid-like form; however, the resonant zwitterionic structure is likely to be dominated upon excitation. A charge transfer character of the first excited state explains the experimentally observed large Stokes shift.

We have also characterized the neutral radical and anionic triplet states of the chromophore inside the S65T GFP protein. The VEEs of the radical form are 833 nm (with an oscillator strength of 0.06), 554 nm (0.02), and 518 nm (0.2). The anionic chromophore in the triplet state most strongly absorbs at around 900 and 500 nm, which does not exclude its formation during the photoconversion, e.g., (Byrdin et al., 2018); however, even if it is formed, the subsequent transformations include the radical chromophore formed either directly upon excitation or indirectly via the triplet state.

**FIGURE 8:**
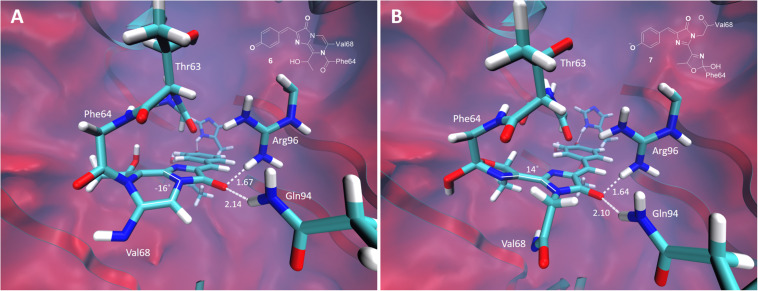
Ground-state equilibrium structures of the cyclic oxidized chromophores. **(A)** The neutral orange-emitting chromophore. **(B)** The red-emitting mOrange-like chromophore. Only the QM parts are shown for clarity. All distances are shown in Å.

The green-emitting quinoid-like structure, if stabilized, can also undergo subsequent cyclization reactions, yielding, for example, the mOrange-like chromophore (Subach and Verkhusha, 2012). Alternatively, the other type of cyclization, with the nitrogen atom acting as a nucleophile, may lead to a new type of the chromophore with two condensed rings. The equilibrium structures of these two types of the chromophores are shown in Figure 8. Both structures are non-planar in the ground electronic state. There is one major distinction between them: the mOrange-like chromophore is formed in its anionic form upon cyclization and deprotonation, whereas the new type of the chromophore remains neutral and is formed upon cyclization and dehydration. The latter exhibits an extended π-conjugated system and a quinoid-like structure similar to that of the parent green-emitting form.

The calculated VEE of the mOrange-like chromophore is 535 nm with an oscillator strength of 0.98. This value is consistent with the experimental absorption maximum of the red-emitting form observed in the photoconversion under the low-oxygen conditions (absorption 535 nm, emission 600 nm). According to the calculations, the first bright transition of the bicyclic structure is at 480 nm with an oscillator strength of 0.53. This corresponds to the absorption maximum of the orange-emitting form observed experimentally at around 480–500 nm. In this case, the large Stokes shift (absorption ∼490 nm, emission 565 nm) can also be traced to the quinoid-like structure of the bicyclic chromophore in the ground state and to the formation of the predominant zwitterionic form in the excited state.

As a result, the red- and orange-emitting forms observed upon photoconversion under the low-oxygen conditions can be attributed to the anionic cyclic and neutral bicyclic structures of the oxidized GFP chromophore, respectively. We note that the bicyclic structure can further be hydrolyzed, which is accompanied by cleavage of the peptide bond. Upon deprotonation of the hydrolyzed structure, the anionic far-red absorbing chromophore is formed with the calculated VEE of 630 nm. This far-red absorbing and emitting chromophore has not been identified experimentally, indicating that the cleavage does not happen within the timeframe of the experiment.

## Discussion

In the current work, we have established conditions for efficient green-to-red photoconversion of EGFP in the absence of molecular oxygen, in agreement with the original report by Elowitz et al. (1997). The red fluorescence lifetime of 2 ns observed upon photoconversion of EGFP is in good agreement with the previous reports (2.1 ns) on green-to-red photoconversion of S65T GFP under low-oxygen conditions in the mitochondrial matrix of living budding yeast (Jakobs et al., 2003). The observed absorption and excitation spectra with a maximum at 535 nm are also in agreement with the value obtained by differential excitation spectroscopy (Elowitz et al., 1997). Similarly, the red-emitting forms found in the current work are stable for at least tens of minutes, in agreement with all other reports on low-oxygen GFP photoconversion (Elowitz et al., 1997; Jakobs et al., 2003) and in strike contrast with the unstable red-emitting product of oxidative photoconversion of EGFP (Bogdanov et al., 2009b). The fluorescence lifetime of the product of oxidative photoconversion is for the first time measured in the present work and equals ∼0.6 ns. Such an extremely short lifetime suggests that different structures of the chromophore are responsible for the appearance of red emission following oxidative and low-oxygen photoconversions.

Another type of green-to-red photoconversion is known for fluorescent proteins sharing His-Tyr-Gly chromophore-forming triad, first discovered in the Kaede protein from a stony coral (Ando et al., 2002). This type of photoconversion is efficiently induced by UV (Ando et al., 2002) in all of these proteins, and, in some cases, by blue (Gurskaya et al., 2006), or a combination of blue and red light (Dempsey et al., 2015). Importantly, the molecular brightness of red-emitting species can reach the values typical for conventional red fluorescent proteins. Moreover, the photoconversion efficiency typically exceeds 50% (Durisic et al., 2014), making Kaede-like proteins popular tools for super-resolution microscopy. In contrast, red-emitting species that appear in the course of oxidative redding (Bogdanov et al., 2009b) are dim (quantum yield ∼0.05). Interestingly, the photon counts detected from EGFP and red-emitting EGFP species after the photoconversion with reduced flavins were on the same scale in the super-resolution setup (Matsuda et al., 2010). The exact molecular brightness of red-emitting EGFP species and the maximum photoconversion efficiency of EGFP under various illumination intensities remains to be elucidated.

While the experimental conditions used for the green-to-red photoconversion in this study may be considered “reductive,” it is hard to imagine a red-shifted version of the GFP chromophore which is not formed as a result of some type of oxidation. Moreover, the computational studies suggest that side chains of certain amino acid residues in the immediate vicinity of the chromophore may serve as primary electron acceptors in photoinduced electron transfer (Bogdanov et al., 2016). Overall, the first step in both oxidative and low-oxygen redding is likely a result of the chromophore oxidation, which suggests that both types of green-to-red photoconversions of GFP may share common intermediates.

**FIGURE 9:**
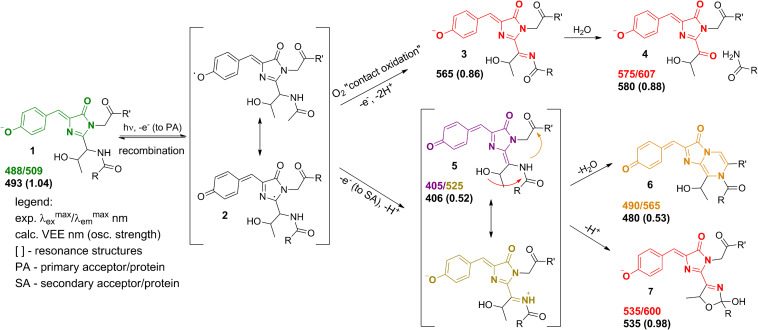
Proposed mechanism of the green-to-red GFP photoconversions. The molecular species correspond to **(1)** – the native GFP chromophore, **(2)** – the radical state, **(3,4)** – the red-emitting forms in oxidative redding, **(5)** – the green-emitting quinoid-like form detected upon photoconversion under the low-oxygen conditions, **(6,7)** – the orange and red-emitting photoproducts observed under the low-oxygen conditions.

Experimentally, three emitting states have been identified upon the low-oxygen photoconversion of EGFP in the present work. These species have distinct spectroscopic properties: the green-emitting form with λ_*abs*_/λ_*em*_ = 405/525 nm, the orange-emitting form with λ_*abs*_/λ_*em*_ = 490/565 nm, and the red-emitting form with λ_*abs*_/λ_*em*_ = 535/600 nm. The red-emitting form in oxidative redding is characterized by λ_*abs*_/λ_*em*_ = 575/607 nm (Bogdanov et al., 2009b). Our calculated VEEs are in good agreement with the observed absorption/excitation maxima, thus providing a ground for assigning the observed experimental peaks (see Figure 9): structure **5** (406 nm) to the green-emitting state, structure **6** (480 nm) to the orange-emitting state, and structure **7** (535 nm) to the red-emitting chromophore detected upon photoconversion under the low-oxygen conditions. Structure **3** (565 nm) and, in particular, structure **4** (580 nm) can be assigned to the photoproduct in oxidative redding. Based on these results, we propose a general mechanism for various green-to-red photoconversions of EGFP.

To the best of our knowledge, the green-emitting product (excitation/emission maxima at 405/525 nm) has not been previously reported. Yet, the prominent rise and fall of its emission in a light-independent manner, accompanied by a subsequent rise of orange emission at 565 nm, suggests an important role of the 525-nm emitting form as an intermediate in conversion to the final photoproducts. Our computational results reveal the neutral quinoid-like form of the chromophore as a most likely candidate for this unusual green-emitting form (Figure 9, structure **5**). Its light-independent interconversion leads to the orange-emitting (Figure 9, structure **6**) as well as red-emitting (Figure 9, structure **7**) chromophores. These structures are formed following two different types of cyclization reactions, which may compete with each other. The delayed appearance of the 565 nm emitting form can be explained by a two-step process, where cyclization is followed by dehydration. At the same time, the red-emitting form appears promptly following the mOrange-like cyclization of the chromophore (Subach and Verkhusha, 2012). Importantly, the latter process may only occur in the case of GFP with Ser or Thr at amino acid position 65. Indeed, both orange and red emitting forms are observed upon photoconversion of EGFP, whereas only the orange-emitting state is detected with the T65G EGFP mutant.

The 565 nm emitting form is attributed to the neutral bicyclic oxidized structure and reported here for the first time. Similar to the 525 nm green emitting form, it exhibits a large Stokes shift, which can be traced to the drastic change of its electronic structure upon photoexcitation. The orange-emitting chromophore does not decay for at least 1 h after photoconversion and its stability does not depend on pH. At the same time, the 600 nm emitting chromophore appears to be more stable under high pH and shows considerable decay at pH 6.5 (Figure 3B). This is consistent with the anionic nature of the red-emitting chromophore. Protonation, as well as hydrolysis of the dihydrooxazole ring following its protonation, might be responsible for quenching of this form in the ground electronic state. In contrast, the 565 nm emitting form is neutral and exhibits a quinoid-like structure, therefore, it is indeed not sensitive to variations in pH, supporting our assignment.

Overall, the proposed mechanism (Figure 9) is in good agreement with the published reports on various photoconversions of green fluorescent protein in the presence of redox-active compounds. Specifically, the radical state (**2**) can be reached via photoinduced electron transfer (Bogdanov et al., 2016) or indirectly via quenching of a triplet state (Byrdin et al., 2018) of the chromophore. Our mechanism is based on two-electron oxidation of the chromophore in the formal presence of oxidizing as well as reducing agents. We believe that the role of redox-active compounds, including reductants, is to provide a redox potential for preventing recombination of a radical pair formed following photoexcitation. At the same time, amino acid residues of EGFP located in the vicinity of the chromophore may serve as primary electron acceptors. Lowering oxygen concentration is crucial for the stability of the radical, which otherwise undergoes rapid oxidation through direct interaction with oxygen penetrating the protein barrel. Importantly, the “contact oxidation” also results in the formation of a relatively strong base that deprotonates the oxidized chromophore, directly yielding red forms 3 and 4. In this case, the quinoid-like structure cannot be formed, as well as its cyclic descendants. Previously, it has been shown that EGFP redding with oxidants can be performed under both aerobic and anaerobic conditions, yielding the same photoproducts (Bogdanov et al., 2009b). However, in light of the present findings, it would be worth revisiting these studies on photoconversion under low-oxygen conditions in the presence of oxidants, rather than reductants, since green-to-red photoconversion occurs in both cases, but some intermediates have been discovered only now.

To conclude, we offer a solution to the long-standing enigma on the appearance of the red-emitting forms of the GFP chromophore upon photoconversion in the presence of reducing agents. We show that these species are different from those previously detected in oxidative redding. Based on our combined experimental and computational studies, we clearly distinguish between various forms and assign them to particular structures of the emitting species. The stabilization/destabilization of the newly discovered green-emitting chromophore (structure **5** in Figure 9) is thought to be a key point in photoconversion proceeding along one pathway or the other. This is confirmed experimentally, where the novel green-emitting state is only observed under low-oxygen conditions in the presence of reductants.

## Data Availability Statement

The datasets generated for this study are available on request to the corresponding author.

## Author Contributions

AM and KL devised the project. DG, AMB, and AM performed photoconversion experiments and steady-state spectroscopy. AM, KL, AMB, MB, and AVB analyzed the data. EM performed time-resolved fluorescence spectroscopy and analysis. AK, EP, and AVB performed computational studies. AM, MB, AK, and AVB devised the proposed mechanism of the photoconversion. AM and AVB wrote the manuscript. All the authors exchanged comments during the manuscript preparation.

## Conflict of Interest

The authors declare that the research was conducted in the absence of any commercial or financial relationships that could be construed as a potential conflict of interest.
